# Associations of Self-Reported Temporomandibular Disorder Symptoms with Sleep Problems, Perceived Stress, and Alexithymia in Young Adults: A Cross-Sectional Study

**DOI:** 10.3390/jcm15218272

**Published:** 2026-10-23

**Authors:** Gökçe Bağcı Uzun, Hakan Ocak, Ayla Arslan, Halil Yılmaz, Emre Demirel, Muhammed Furkan Arpacı, Murat Ayhan, Hıdır Pekmez

**Affiliations:** 1 Department of Anatomy, Faculty of Medicine, Malatya Turgut Özal University, Malatya 44210, Turkey; furkan.arpaci@ozal.edu.tr (M.F.A.); hidir.pekmez@ozal.edu.tr (H.P.); 2 Department of Anatomy, Faculty of Medicine, Ağrı Ibrahim Cecen University, Ağrı 04100, Turkey; hocak@agri.edu.tr (H.O.); aaylaarslan@gmail.com (A.A.); 3 Department of Anatomy, Faculty of Medicine, Ordu University, Ordu 52200, Turkey; halilyilmaz855@gmail.com; 4 Department of Anatomy, Faculty of Medicine, Erciyes University, Kayseri 38039, Turkey; fzt.emredemirel@gmail.com; 5 Malatya Provincial Directorate of Youth Sports, Ministry of Youth and Sports, Malatya 44070, Turkey; muayhan44@gmail.com

**Keywords:** temporomandibular disorders, alexithymia, perceived stress, sleep, young adults

## Abstract

**Background:** Temporomandibular disorders (TMDs) are increasingly understood as chronic pain conditions in which biological susceptibility and psychosocial factors interact. Alexithymia, a personality trait characterized by difficulty identifying and describing feelings, has been linked to chronic pain; however, its relationship with self-reported TMD symptoms, perceived stress, and sleep problems in non-clinical young adults remains insufficiently explored. **Objective:** To examine the associations of self-reported TMD symptom severity with sleep problems, perceived stress, and alexithymia in young adults. **Methods:** In this cross-sectional online survey, 634 participants aged 18–40 years (74.3% female) completed the Fonseca Anamnestic Index (FAI), the 20-item Toronto Alexithymia Scale (TAS-20), the 14-item Perceived Stress Scale (PSS-14), and the Jenkins Sleep Scale (JSS). Group comparisons, Spearman correlations, and hierarchical multiple linear regression with FAI score as the dependent variable were performed. All analyses were re-run on the raw data during revision. **Results:** Self-reported TMD symptoms of any severity were present in 90.5% of participants. FAI scores correlated weakly to moderately with JSS (r = 0.40), PSS-14 (r = 0.37), and TAS-20 (r = 0.29) scores (all *p* < 0.001). After adjustment for sociodemographic variables (R^2^ = 0.147), sleep problems (β = 0.24), perceived stress (β = 0.16), and alexithymia (β = 0.14) explained an additional 14.6% of the variance in FAI scores (final R^2^ = 0.292; *p* < 0.001). Female sex, older age within the 18–40 range, and residence in an earthquake-affected province were also independently associated with higher FAI scores. Among the alexithymia dimensions, only difficulty identifying feelings was independently associated with FAI scores. **Conclusions:** In this young adult sample, self-reported TMD symptom severity was associated with sleep problems, perceived stress, and alexithymia, with weak-to-moderate effect sizes. Given the cross-sectional design and reliance on self-report, these findings reflect associations only—causal or temporal relationships cannot be inferred, and the findings support considering psychosocial factors when TMD-related symptoms are assessed.

## 1. Introduction

Temporomandibular disorders (TMDs) represent a heterogeneous group of musculoskeletal and neuromuscular conditions involving the temporomandibular joints (TMJs), the masticatory muscles, and associated structures [1,2]. Pain may be perceived in the TMJ region, the masticatory muscles, and the preauricular area, and may extend to the head, neck, face, and ear. Other common features include limited or altered mandibular movement and joint sounds during function [2,3]. Although TMDs affect only 5–10% of the population, related symptoms are reported by up to 50% of adults [3]. Current evidence conceptualizes painful TMD as a chronic pain condition: prospective data from the OPPERA studies indicate that its onset and persistence reflect a complex interplay of genetic susceptibility, altered central pain processing, and psychosocial factors, rather than a single local cause [4,5]. Within this framework, articular and muscular findings, as well as psychosocial characteristics such as depression, somatization, and stress, are best regarded as associated or modifying factors rather than as etiologies [3,4].

TMDs are multifactorial. Local factors such as occlusal features and tooth-contact habits have long been discussed, although current consensus statements indicate that dental occlusion plays at most a minor role [3,6]. Systemic health and medication use may also influence symptom severity; for example, the pharmacotherapy of general diseases has been examined in relation to TMD symptoms [7]. Epidemiological studies consistently show that TMDs are more common in women than in men, with a female-to-male ratio of approximately 2:1, and that prevalence peaks in young and middle adulthood (approximately 20–40 years) [8,9]. Young adults, including university students, frequently face academic, financial, and interpersonal stressors [10]. These stressors are common in this age group and are not in themselves causes of TMD, but young adults are a relevant population in which to study TMD-related symptoms and their psychosocial correlates before chronic pain patterns become established [11,12].

Sleep is an active, regulated process consisting of rapid eye movement (REM) sleep and non-rapid eye movement (NREM) sleep. According to current terminology, NREM sleep is subdivided into stages N1 (transition from wakefulness), N2 (characterized by sleep spindles and K-complexes), and N3 (slow-wave sleep, characterized by delta activity) [13,14]. Disturbed sleep alters the secretion patterns of hormones such as growth hormone, cortisol, and testosterone [15] and is associated with anxiety, depression, and poorer mental health [16,17,18]. Poor sleep is common in young adults. In a large multi-university sample, 62% of students met criteria for poor sleep quality [19]. Prospective data also indicate that subjective sleep quality deteriorates before the onset of painful TMD [20], and a recent systematic review supports an association between sleep disorders and TMD in adults [21].

Alexithymia is a multidimensional personality construct characterized by difficulty identifying feelings (DIF), difficulty describing feelings (DDF), and an externally oriented thinking style (EOT) [22,23,24]. It is not a medical or psychiatric diagnosis and is not classified as a mental disorder in the DSM-5; rather, it is regarded as a dimensional trait that may increase vulnerability to a range of medical and psychiatric conditions [22,23]. Alexithymia occurs in approximately 10% of the general population, with prevalence varying by sex, age, socioeconomic status, and comorbidity [25]. It has been associated with panic disorder, generalized anxiety disorder, and major depressive disorder [26], with sleep problems [27], and with chronic craniofacial pain [28,29].

Previous studies have examined pairs of these variables—for example, alexithymia and sleep [27], alexithymia and craniofacial pain [28], and stress, sleep, and TMD symptoms in dental students [11,12,30]. However, to our knowledge, no study has examined self-reported TMD symptoms, perceived stress, sleep problems, and alexithymia simultaneously in a non-clinical young adult sample, while also estimating how much these psychosocial variables contribute beyond sociodemographic factors. In addition, most student studies have been limited to dental students, and few have considered the 6 February 2023 Kahramanmaraş earthquakes, which affected a large proportion of the population in the study region. Participants in the present study were not recruited from a clinical TMD population and were not required to report TMD-related complaints.

The aim of this study was, therefore, to examine the associations of self-reported TMD symptom severity with sleep problems, perceived stress, and alexithymia in 18–40-year-old adults. We hypothesized that (H1) higher FAI scores would be associated with more sleep problems, higher perceived stress, and higher alexithymia, (H2) these psychosocial variables would explain variance in FAI scores beyond sociodemographic and health-related variables, and (H3) among the alexithymia dimensions, the affective components (DIF and DDF) rather than EOT would be associated with TMD symptoms.

## 2. Materials and Methods

### 2.1. Study Design and Sample Size

This was a cross-sectional, questionnaire-based study reported in accordance with the STROBE guidelines for cross-sectional studies. An a priori sample size calculation was performed with G*Power 3.1 for multiple linear regression (fixed model, R^2^ deviation from zero) with 12 predictors, a medium effect size (f^2^ = 0.15), α = 0.05, and a statistical power of 0.95 (1−β)—the minimum required sample size was 184. In the original submission the power value was erroneously labeled as a “confidence interval”. A sensitivity analysis showed that the final sample (n = 634) provided 95% power to detect an increase in R^2^ corresponding to f^2^ ≥ 0.027 (a small effect) for the block of three psychosocial predictors.

### 2.2. Participants and Recruitment

Data were collected via an anonymous online questionnaire (Google Forms) between 26 January 2024 and 20 March 2024. A convenience sampling approach was used: the survey link was distributed via WhatsApp groups to university students and young adults in the Malatya, Ağrı, Ordu, Kayseri, and Elazığ provinces of Turkey, and participants were encouraged to share the link with peers. Inclusion criteria were age 18–40 years and electronic approval of the informed consent statement, which was required before the questionnaire could be accessed. Because the survey was anonymous and self-administered and contained no item on previous maxillofacial surgery or dependence on personal care, these originally listed exclusion criteria could not be verified, and this is acknowledged as a limitation. In the original submission, a history of psychiatric diagnosis was erroneously listed as an exclusion criterion. Psychiatric status could not be clinically verified; instead, participants were asked, “Do you have a psychological disorder?” (yes/no). Respondents answering “yes” (n = 71) were retained, and this self-reported variable (hereafter “self-reported psychological complaint”) was entered as a covariate. A sensitivity analysis excluding these participants was also performed.

A total of 729 responses were received. During the re-analysis of the raw data for this revision, respondents with missing age (n = 1), those younger than 18 years (n = 10), and those older than 40 years (n = 84) were excluded in accordance with the inclusion criteria, leaving 634 participants for analysis (Table 1). The original submission had erroneously included respondents outside the 18–40-year range. Sociodemographic data included age, sex, height, weight, place of birth, family socioeconomic status, chronic disease, self-reported psychological complaint, smoking, and the province of residence during the 6 February 2023 earthquakes. Body mass index (BMI) was calculated from self-reported height and weight, and implausible values (height < 140 or > 210 cm; weight < 35 or > 200 kg) were treated as missing. Residence in an earthquake-affected province was defined as residence in 1 of the 11 provinces officially declared disaster areas (Adana, Adıyaman, Diyarbakır, Elazığ, Gaziantep, Hatay, Kahramanmaraş, Kilis, Malatya, Osmaniye, and Şanlıurfa). The complete questionnaire is provided in Supplementary File S1.

### 2.3. Ethical Considerations

The study was approved by the Malatya Turgut Özal University Non-Interventional Clinical Research Ethics Committee (date: 20 January 2024; Decision No. 2024/38) and was conducted in accordance with the Declaration of Helsinki. No identifying information was collected, and participants were informed about available counselling services.

### 2.4. Measures

*Fonseca Anamnestic Index (FAI).* The FAI is a 10-item self-report questionnaire developed to screen for TMD-related symptoms and to classify their perceived severity [31]. Items address difficulty opening the mouth, difficulty moving the mandible sideways, muscle fatigue or pain during chewing, frequent headaches, neck pain or stiffness, ear or TMJ pain, joint clicking, tooth clenching or grinding, the perception of an altered dental occlusion, and self-perceived nervousness. Each item is answered “yes” (10 points), “sometimes” (5 points), or “no” (0 points). Total scores range from 0 to 100 and are classified as no TMD (0–15), mild (20–40), moderate (45–65), and severe (70–100). The validity and reliability of the Turkish version have been demonstrated [32]. The FAI is a screening tool and does not establish a clinical diagnosis [33]; therefore, throughout this manuscript, FAI scores are referred to as “self-reported TMD symptom severity”.

*Toronto Alexithymia Scale (TAS-20).* The TAS-20 consists of 20 items rated on a 5-point Likert scale (1 = strongly disagree to 5 = strongly agree) [24]. Items 4, 5, 10, 18, and 19 are reverse scored, and total scores range from 20 to 100. The scale has three subscales: DIF (items 1, 3, 6, 7, 9, 13, and 14), DDF (items 2, 4, 11, 12, and 17), and EOT (items 5, 8, 10, 15, 16, 18, 19, and 20). Scores ≤ 51 indicate no alexithymia, 52–60 borderline alexithymia, and ≥ 61 alexithymia [24]. The Turkish version has been validated [34].

*Jenkins Sleep Scale (JSS).* The JSS is a four-item scale assessing the frequency of difficulty falling asleep, waking up several times during the night, difficulty staying asleep (including waking up too early), and waking up feeling tired and worn out after the usual amount of sleep during the previous month [35]. Each item is scored from 0 to 5 (0 = not at all, 1 = 1–3 days, 2 = 4–7 days, 3 = 8–14 days, 4 = 15–21 days, and 5 = 23–31 days per month), giving a total score of 0–20—higher scores indicate more sleep problems. The Turkish version has been validated [36].

*Perceived Stress Scale (PSS-14).* The PSS-14 measures the degree to which situations in one’s life during the previous month are appraised as unpredictable, uncontrollable, and overloading [37]. Its 14 items are rated on a 5-point scale (0 = never to 4 = very often). The seven positively worded items (4, 5, 6, 7, 9, 10, and 13) are reverse scored, and total scores range from 0 to 56, with higher scores indicating greater perceived stress. The Turkish version has been validated in university students [38]. In the original submission, scores had inadvertently been calculated from the 10-item subset (PSS-10). All analyses in this revision use the full 14-item scale that was administered, and results based on the PSS-10 are reported as a sensitivity analysis.

### 2.5. Statistical Analysis

During revision, all scale scores were recalculated from the raw item-level data, and all analyses were repeated. This re-analysis identified that the reverse-scored TAS-20 items had not been reversed in the original analysis, and this error has been corrected throughout. Analyses were performed in Python 3 using the NumPy and SciPy libraries. Normality was assessed with the Kolmogorov–Smirnov test. Because the distributions of the FAI, PSS-14, and JSS scores deviated from normality (*p* < 0.05), non-parametric tests were used for all bivariate analyses. The Mann–Whitney U test was used for comparisons between two independent groups and the Kruskal–Wallis test analysis was used for comparisons among more than two groups, with Dunn’s post hoc test and Bonferroni correction for pairwise comparisons. Effect sizes are reported as r (z/√N) for the Mann–Whitney U test and epsilon-squared (ε^2^) for the Kruskal–Wallis test. Associations between continuous variables were assessed with Spearman’s rank correlation coefficient and interpreted as weak (|r| = 0.10–0.29), moderate (0.30–0.49), or strong (≥0.50). The association between FAI and TAS-20 categories was examined with the chi-square test. Internal consistency was assessed with Cronbach’s α. Statistical significance was set at *p* < 0.05 (two-tailed).

Hierarchical multiple linear regression was performed with the FAI total score (0–100) as the dependent variable. Model 1 included sociodemographic and health-related variables: sex (female = 1, male = 0), age (years), BMI (kg/m^2^), family socioeconomic status (two dummy variables, low and high, with middle as the reference category), chronic disease (yes = 1), self-reported psychological complaint (yes = 1), smoking (yes = 1), and residence in an earthquake-affected province (yes = 1). Model 2 additionally included PSS-14, JSS, and TAS-20 total scores. The change in R^2^ (ΔR^2^) between the models was tested with the F-change test. Unstandardized (B) and standardized (β) coefficients with 95% confidence intervals are reported. Because ordinary least squares regression does not require normally distributed predictors, and to account for possible heteroscedasticity, heteroscedasticity-consistent (HC3) standard errors were used. Multicollinearity was assessed with variance inflation factors (VIF), and residuals were inspected for normality. Missing BMI values (n = 28) were replaced with the sample median. Five sensitivity analyses were performed: (i) replacing the TAS-20 total score with its three subscales, (ii) using the PSS-10 instead of the PSS-14, (iii) including all respondents aged ≥ 18 years (n = 718), (iv) excluding participants with a self-reported psychological complaint (n = 563), and (v) excluding the FAI nervousness item (item 10) from the dependent variable to reduce conceptual overlap with perceived stress.

## 3. Results

### 3.1. Sample Characteristics

Of the 634 participants, 471 (74.3%) were female and 163 (25.7%) were male. The mean age was 25.98 ± 6.41 years (range 18–40), and the mean BMI was 23.67 ± 4.41 kg/m^2^. Most participants reported a middle family socioeconomic status (85.5%). A chronic disease was reported by 70 (11.0%) participants, a psychological complaint by 71 (11.2%), and current smoking by 218 (34.4%), and 480 (75.7%) participants had resided in an earthquake-affected province on 6 February 2023 (Table 1).

### 3.2. Scale Scores and TMD Symptom Severity

The mean FAI, TAS-20, PSS-14, and JSS scores were 49.91 ± 23.88, 55.90 ± 10.46, 31.23 ± 7.37, and 10.17 ± 5.05, respectively (Table 2). According to the FAI, 60 participants (9.5%) had no TMD symptoms, whereas 195 (30.8%), 207 (32.6%), and 172 (27.1%) reported mild, moderate, and severe symptoms, respectively. Based on the TAS-20, 227 participants (35.8%) were classified as alexithymic and 176 (27.8%) as borderline. Internal consistency was acceptable to good for all scales (Cronbach’s α = 0.70–0.85) except the EOT subscale (α = 0.28). PSS-14 (H = 88.28, ε^2^ = 0.139), JSS (H = 98.04, ε^2^ = 0.155), and TAS-20 (H = 61.26, ε^2^ = 0.097) scores increased across FAI severity categories (all *p* < 0.001; Table 3). Among the TAS-20 subscales, DIF (H = 88.69, ε^2^ = 0.140) and DDF (H = 32.75, ε^2^ = 0.052) scores differed across FAI categories (both *p* < 0.001), whereas EOT scores did not (H = 1.71, *p* = 0.636). FAI and TAS-20 categories were significantly associated (χ^2^(6) = 60.73, *p* < 0.001; Supplementary Table S3).

### 3.3. Correlations Among Study Variables

FAI scores were moderately correlated with JSS (r = 0.40) and PSS-14 (r = 0.37) scores and weakly correlated with TAS-20 scores (r = 0.29; all *p* < 0.001; Table 4). Among the TAS-20 subscales, FAI scores correlated with DIF (r = 0.35, *p* < 0.001) and DDF (r = 0.20, *p* < 0.001), but not with EOT (r = 0.03, *p* > 0.05). PSS-14 scores correlated moderately with JSS (r = 0.42) and TAS-20 (r = 0.46) scores, and JSS and TAS-20 scores were moderately correlated (r = 0.32; all *p* < 0.001). Age was weakly and negatively correlated with PSS-14 (r = −0.22) and TAS-20 (r = −0.19) scores (both *p* < 0.001), but not with FAI scores (r = 0.04). BMI was not correlated with FAI scores.

### 3.4. Scale Scores According to Sociodemographic Variables

Women had higher FAI (53.93 ± 22.78 vs. 38.28 ± 23.23; *p* < 0.001, r = 0.28), PSS-14 (*p* < 0.001, r = 0.21), and JSS (*p* < 0.001, r = 0.15) scores than men, and slightly higher TAS-20 scores (*p* = 0.017, r = 0.09; Table 5). Participants with a self-reported psychological complaint had higher scores on all four scales (FAI *p* = 0.019; PSS-14, JSS, and TAS-20 *p* < 0.001), as did smokers (all *p* < 0.05).

Participants who had resided in an earthquake-affected province had higher FAI (52.01 ± 24.36 vs. 43.34 ± 21.10; *p* < 0.001, r = 0.16), PSS-14 (*p* = 0.001), and JSS (*p* < 0.001) scores, whereas TAS-20 scores did not differ (*p* = 0.157). Chronic disease was not associated with any scale score (all *p* > 0.05).

Family socioeconomic status was associated only with TAS-20 scores (H = 11.40, *p* = 0.003), with the highest socioeconomic group showing the lowest scores—it was not associated with FAI, PSS-14, or JSS scores (all *p* > 0.05; Table 5). All effect sizes for group differences were small (r ≤ 0.28).

### 3.5. Hierarchical Regression Analysis

Model 1, including sociodemographic and health-related variables, explained 14.7% of the variance in FAI scores (R^2^ = 0.147, adjusted R^2^ = 0.134; F(9, 624) = 11.92, *p* < 0.001). Adding PSS-14, JSS, and TAS-20 scores in Model 2 explained an additional 14.6% of the variance (ΔR^2^ = 0.146; F-change(3, 621) = 42.64, *p* < 0.001), resulting in a final R^2^ of 0.292 (adjusted R^2^ = 0.279; F(12, 621) = 21.39, *p* < 0.001; Table 6). In Model 2, sleep problems (JSS: B = 1.14, β = 0.24, *p* < 0.001), perceived stress (PSS-14: B = 0.53, β = 0.16, *p* < 0.001), and alexithymia (TAS-20: B = 0.31, β = 0.14, *p* = 0.002) were independently associated with higher FAI scores. Being female was associated with FAI scores that were 12.89 points higher than those of males (95% CI 8.65–17.14; β = 0.24, *p* < 0.001). Older age within the 18–40-year range (B = 0.52 per year, β = 0.14, *p* < 0.001) and residence in an earthquake-affected province (B = 5.49, β = 0.10, *p* = 0.004) were also associated with higher FAI scores. Self-reported psychological complaint (*p* = 0.993), smoking (*p* = 0.052), chronic disease, socioeconomic status, and BMI were not independently associated with FAI scores. All VIF values were below 1.6, and the residuals were approximately normally distributed (skewness = 0.02; excess kurtosis = −0.51).

When the TAS-20 total score was replaced by its subscales, only DIF was independently associated with FAI scores (β = 0.22, *p* < 0.001), whereas DDF (β = −0.02, *p* = 0.661) and EOT (β = −0.05, *p* = 0.162) were not (Supplementary Table S1). The associations of JSS, PSS-14, and TAS-20 scores with FAI scores remained significant and of similar magnitude in all sensitivity analyses: using the PSS-10 (R^2^ = 0.291), including all respondents aged ≥ 18 years (n = 718; R^2^ = 0.290), using the original sample of 728 respondents (R^2^ = 0.285), excluding participants with a self-reported psychological complaint (n = 563; R^2^ = 0.301), and excluding the FAI nervousness item (R^2^ = 0.262; Supplementary Table S2).

## 4. Discussion

This study examined the associations of self-reported TMD symptom severity with sleep problems, perceived stress, and alexithymia in 634 young adults. In line with our hypotheses, higher FAI scores were associated with more sleep problems, higher perceived stress, and higher alexithymia (H1), and these three psychosocial variables explained an additional 14.6% of the variance in FAI scores beyond sociodemographic and health-related variables (H2). Consistent with H3, the association with alexithymia was driven by difficulty identifying feelings, whereas externally oriented thinking was not associated with TMD symptoms. The effect sizes were weak to moderate, and the final model explained 29% of the variance, indicating that most of the variability in self-reported TMD symptoms is attributable to factors not measured in this study.

Of the three psychosocial variables, sleep problems showed the strongest independent association with FAI scores. This finding is consistent with prospective OPPERA data showing that poor subjective sleep quality precedes the onset of painful TMD [20] and with a systematic review supporting an association between sleep disorders and TMD [21]. Because the relationship between sleep and pain is considered bidirectional, and because our data are cross-sectional, we cannot determine whether sleep problems contribute to TMD symptoms, result from them, or both reflect a shared underlying vulnerability.

Perceived stress was also independently associated with FAI scores. Unresolved perceived stress can manifest through psychological, cognitive, and somatic symptoms [39] and has been associated with poorer perceived health and with somatic conditions [40,41]. Stress has also been linked to TMD symptoms in several student populations [11,12,30] and to stress-related behaviors in individuals with TMJ dysfunction [42], and it may be related to oral parafunctional behaviors, such as awake clenching [43,44]. However, oral behaviors were not measured in the present study, and these pathways remain speculative. Similarly, alexithymia was independently, albeit weakly, associated with FAI scores. The subscale analysis showed that this association was specific to DIF, which is in line with previous findings that DIF—rather than EOT—is the alexithymia component most consistently associated with chronic pain, anxiety, and depression [28,29], and that individuals with high DIF scores report higher perceived stress [45]. The very low internal consistency of the EOT subscale (α = 0.28) in our sample means that the null finding for EOT should be interpreted with caution.

These findings are best interpreted within the current understanding of painful TMD as a chronic pain condition [4,5]. According to this view, TMD develops and persists through the interaction of genetic susceptibility, altered central pain processing (for example, central sensitization and dysfunction of pain-modulating neural networks), and psychosocial factors [4,5]. Neuroimaging studies of alexithymia have shown altered activity in regions involved in emotion processing, including the anterior cingulate cortex, amygdala, and insula [46,47], which overlap with regions involved in pain processing. Sleep problems, perceived stress, and alexithymia may, therefore, be understood as psychosocial correlates or modifying factors within this multisystem framework, rather than as etiological factors. Importantly, many young adults experience stress and sleep problems without developing TMD symptoms, which further indicates that these factors are neither necessary nor sufficient causes.

Bruxism deserves specific consideration. According to the international consensus, bruxism is a repetitive jaw-muscle activity with two distinct circadian manifestations—sleep bruxism and awake bruxism—and is regarded as a behavior that may be a risk factor or a protective factor for health outcomes, rather than a disorder in itself [48]. In our sample, 67.8% of participants answered “yes” or “sometimes” to the FAI item on clenching or grinding. The literature on bruxism and alexithymia is inconsistent: some studies have reported a positive association in TMD patients [49], whereas no relationship was found in women with sleep bruxism [50]. Awake bruxism has been more consistently linked to alexithymia, anxiety, and perceived stress [43]. Because the FAI includes only a single item on clenching or grinding and does not distinguish between sleep and awake bruxism, we could not examine bruxism as a separate variable. Future studies should assess both types of bruxism with validated instruments.

The sex difference in our sample was substantial: women scored on average 12.9 points higher on the FAI than men after adjustment for the other variables. This is consistent with epidemiological evidence that TMDs are approximately twice as common in women as in men [8,9], a difference that has been attributed to biological factors, such as hormonal influences on pain sensitivity, as well as to psychosocial factors and differences in healthcare seeking [4,9]. Age was positively associated with FAI scores in the adjusted model, which is consistent with the reported increase in TMD prevalence from adolescence to a peak in young and middle adulthood [8]. In contrast, age was negatively associated with perceived stress and alexithymia, suggesting that emotional awareness and coping may improve with age in this age range.

Participants who had resided in an earthquake-affected province reported higher FAI, perceived stress, and sleep problem scores, and residence in these provinces remained independently associated with FAI scores in the adjusted model. This finding should be interpreted cautiously: earthquake exposure was defined only by the province of residence, and trauma-related symptoms, depression, anxiety, bereavement, and displacement were not assessed. These unmeasured factors may confound the observed associations. Smoking and self-reported psychological complaints were associated with higher scores in bivariate analyses, but not after adjustment for perceived stress, sleep problems, and alexithymia, suggesting that their bivariate associations with TMD symptoms may be largely accounted for by these psychosocial variables.

From a clinical perspective, our findings support the recommendation of current consensus statements that the assessment of patients with TMD-related complaints should include psychosocial factors (Axis II of the DC/TMD) in addition to physical findings [6,51,52]. Management of TMD is primarily conservative and includes patient education, self-management, physiotherapy, pharmacotherapy, and psychological approaches, such as cognitive behavioral therapy [3,6]. Occlusal splints are widely used; however, a recent systematic review found insufficient evidence that occlusal splint therapy is superior to other interventions or no treatment for orofacial myalgia and myofascial pain [53]. It has also been suggested that peripherally targeted treatments, such as splints, may be more effective in patients with localized pain than in those with more widespread, centralized pain [5]. Because the present study did not evaluate any intervention, these considerations are offered as context rather than as conclusions drawn from our data.

When interpreting these findings, it should be borne in mind that the FAI is a self-report screening tool rather than a clinical diagnostic instrument [33]. Accordingly, the high proportion of participants with mild-to-severe scores (90.5%) reflects the burden of self-reported TMD-related symptoms rather than the prevalence of clinically diagnosed TMD, and it is likely inflated by the inclusion of non-specific items, such as headache, neck pain, and nervousness. Future studies that combine self-report measures with a standardized clinical examination according to the DC/TMD [52] will help to clarify the clinical significance of these symptoms in young adults.

The strengths of this study include its relatively large sample, which exceeded the required sample size, the use of validated Turkish versions of all instruments, the simultaneous assessment of four related constructs, the adjustment for sociodemographic and health-related variables in a hierarchical model, the analysis of alexithymia subscales, and the consistency of the results across five sensitivity analyses. In addition, all scale scores were recalculated from the raw item-level data during revision, which allowed a scoring error in the original analysis to be identified and corrected.

### Limitations and Future Directions

This study has several limitations. First, the FAI is a self-report screening tool that is not based on clinical examination or validated diagnostic criteria, such as the DC/TMD [33,52]; therefore, no distinction could be made between subtypes, such as myofascial pain and intra-articular disorders, and the findings refer to self-reported TMD symptom severity rather than clinically diagnosed TMD. Second, because the FAI contains items on headache, neck pain, and nervousness, part of its association with perceived stress may reflect conceptual overlap; however, the results were similar when the nervousness item was excluded.

Third, the types of bruxism (sleep and awake) were not assessed separately, and the internal consistency of the TAS-20 EOT subscale was very low (α = 0.28). Fourth, important potential confounders—including depression, anxiety, trauma-related symptoms following the 2023 earthquakes, bruxism, and medication use [7]—were not measured, self-reported psychological complaints were assessed with a single yes/no question, and previous maxillofacial surgery could not be verified as an exclusion criterion.

Fifth, convenience sampling through an online survey distributed via WhatsApp groups (which also precluded the calculation of a response rate), the predominance of women (74.3%), and the regional sample limit the generalizability of the findings to the general population, to other regions, and to patients with clinically diagnosed TMD. Sixth, all variables were measured with self-report questionnaires at a single time point, so common-method bias may have inflated the observed associations [54].

Finally, owing to the cross-sectional design, the direction of the associations between TMD symptoms, perceived stress, sleep problems, and alexithymia cannot be determined, and the potential mechanisms discussed above (such as awake muscle activity) were not measured. Longitudinal studies combining clinical DC/TMD examination with validated measures of bruxism and psychological distress are needed to clarify these relationships.

## 5. Conclusions

In this cross-sectional study of young adults, self-reported TMD symptom severity was associated with sleep problems, perceived stress, and alexithymia—particularly difficulty identifying feelings—independently of sociodemographic factors. The effect sizes were weak to moderate, and the psychosocial variables explained approximately 15% of the variance in FAI scores. Given the cross-sectional design and reliance on self-reported measures, these findings reflect associations only—causal or temporal relationships, as well as the underlying mechanisms, cannot be inferred. The findings support the consideration of psychosocial factors, including sleep, perceived stress, and emotional awareness, when TMD-related symptoms are assessed in young adults. Longitudinal studies with clinical DC/TMD diagnoses and validated measures of bruxism, depression, anxiety, and trauma are needed.

## Figures and Tables

**Table 1:** Sociodemographic characteristics of the study sample (n = 634).

Variable	Category	n	%
**Sex**	Female	471	74.3
	Male	163	25.7
**Family socioeconomic status**	Low	63	9.9
	Middle	542	85.5
	High	29	4.6
**Chronic disease**	No	564	89.0
	Yes	70	11.0
**Self-reported psychological complaint**	No	563	88.8
	Yes	71	11.2
**Current smoking**	No	416	65.6
	Yes	218	34.4
**Residing in earthquake-affected province (6 Feb 2023)**	No	154	24.3
	Yes	480	75.7
		**Mean ± SD**	**Median (min–max)**
**Age (years)**		25.98 ± 6.41	24 (18–40)
**BMI (kg/m^2^) (n = 606)**		23.67 ± 4.41	23.14 (14.69–46.81)

SD: standard deviation; BMI: body mass index (valid n = 606 after removal of implausible height/weight values).

**Table 2:** Descriptive statistics and internal consistency of the scales (n = 634).

Scale (Possible Range)	Mean ± SD	Median (Min–Max)	Cronbach’s α
**FAI (0–100)**	49.91 ± 23.88	50 (0–100)	0.79
**TAS-20 total (20–100)**	55.90 ± 10.46	56 (30–90)	0.81
**DIF (7–35)**	19.80 ± 6.00	19.5 (7–35)	0.85
**DDF (5–25)**	13.84 ± 3.85	14 (5–25)	0.70
**EOT (8–40)**	22.25 ± 3.53	22 (11–34)	0.28
**PSS-14 (0–56)**	31.23 ± 7.37	31 (9–54)	0.85
**JSS (0–20)**	10.17 ± 5.05	10 (0–20)	0.74
**Category**	**n**	**%**	
**FAI: no TMD (0–15)**	60	9.5	
**FAI: mild (20–40)**	195	30.8	
**FAI: moderate (45–65)**	207	32.6	
**FAI: severe (70–100)**	172	27.1	
**TAS-20: non-alexithymic (≤51)**	231	36.4	
**TAS-20: borderline (52–60)**	176	27.8	
**TAS-20: alexithymic (≥61)**	227	35.8	

SD: standard deviation; FAI: Fonseca Anamnestic Index; TAS-20: Toronto Alexithymia Scale; DIF: difficulty identifying feelings; DDF: difficulty describing feelings; EOT: externally oriented thinking; PSS-14: Perceived Stress Scale; JSS: Jenkins Sleep Scale. FAI categories: no TMD 0–15, mild 20–40, moderate 45–65, and severe 70–100. TAS-20 categories: ≤51 non-alexithymic, 52–60 borderline, and ≥61 alexithymic.

**Table 3:** Scale scores according to self-reported TMD symptom severity (FAI categories; n = 634).

Scale	No TMD (a) n = 60	Mild (b) n = 195	Moderate (c) n = 207	Severe (d) n = 172	H	*p*	ε^2^	Significant Pairs
**PSS-14**	25.90 ± 7.42	29.02 ± 6.59	32.23 ± 6.75	34.38 ± 7.16	88.28	<0.001	0.139	a–b; a–c; a–d; b–c; b–d; c–d
**JSS**	6.65 ± 3.94	8.60 ± 4.63	10.39 ± 4.93	12.93 ± 4.53	98.04	<0.001	0.155	a–c; a–d; b–c; b–d; c–d
**TAS-20**	47.93 ± 10.04	53.99 ± 9.27	57.21 ± 9.41	59.25 ± 11.25	61.26	<0.001	0.097	a–b; a–c; a–d; b–c; b–d
**DIF**	14.22 ± 5.20	18.51 ± 5.40	20.73 ± 5.52	22.10 ± 5.90	88.69	<0.001	0.140	a–b; a–c; a–d; b–c; b–d
**DDF**	11.53 ± 3.79	13.39 ± 3.46	14.29 ± 3.67	14.62 ± 4.13	32.75	<0.001	0.052	a–b; a–c; a–d; b–d
**EOT**	22.18 ± 3.64	22.09 ± 3.31	22.19 ± 3.44	22.53 ± 3.82	1.71	0.636	0.003	–

Values are mean ± SD. H: Kruskal–Wallis statistic; ε^2^: epsilon-squared effect size. Post hoc comparisons: Dunn’s test with Bonferroni correction. The last column lists the pairs of groups that differ significantly (adjusted *p* < 0.05). a: no TMD; b: mild; c: moderate; d: severe.

**Table 4:** Spearman correlation coefficients among study variables (n = 634).

	FAI	PSS-14	JSS	TAS-20	DIF	DDF	EOT	Age
**FAI**	–							
**PSS-14**	0.37 ***	–						
**JSS**	0.40 ***	0.42 ***	–					
**TAS** **-20**	0.29 ***	0.46 ***	0.32 ***	–				
**DIF**	0.35 ***	0.50 ***	0.37 ***	0.90 ***	–			
**DDF**	0.20 ***	0.38 ***	0.23 ***	0.84 ***	0.69 ***	–		
**EOT**	0.03	0.12 **	0.07	0.52 ***	0.22 ***	0.25 ***	–	
**Age**	0.04	−0.22 ***	−0.07	−0.19 ***	−0.24 ***	−0.17 ***	0.04	–
**BMI**	−0.04	−0.10 *	−0.03	−0.04	−0.07	−0.06	0.06	0.37 ***

* *p* < 0.05; ** *p* < 0.01; *** *p* < 0.001. BMI: n = 606. Interpretation: |r| 0.10–0.29 weak; 0.30–0.49 moderate; ≥ 0.50 strong. –, not applicable (diagonal of the correlation matrix).

**Table 5:** Scale scores according to sociodemographic variables (n = 634).

Variable	Group	n	FAI	PSS-14	JSS	TAS-20
**Sex**	Female	471	53.93 ± 22.78	32.15 ± 7.06	10.64 ± 5.04	56.45 ± 10.36
	Male	163	38.28 ± 23.23	28.55 ± 7.63	8.83 ± 4.83	54.29 ± 10.62
	Mann–Whitney U		U = 52,802, *p* < 0.001, r = 0.28	U = 48,836, *p* < 0.001, r = 0.21	U = 45,999, *p* < 0.001, r = 0.15	U = 43,183, *p* = 0.017, r = 0.09
**Chronic disease**	Yes	70	54.29 ± 24.04	31.83 ± 7.33	11.13 ± 5.29	56.74 ± 10.54
	No	564	49.36 ± 23.83	31.15 ± 7.38	10.05 ± 5.01	55.79 ± 10.46
	Mann–Whitney U		U = 22,210, *p* = 0.087, r = 0.07	U = 21,098, *p* = 0.347, r = 0.04	U = 21,954, *p* = 0.125, r = 0.06	U = 20,782, *p* = 0.471, r = 0.03
**Psychological complaint**	Yes	71	55.99 ± 20.56	35.08 ± 8.21	12.48 ± 4.69	60.35 ± 10.71
	No	563	49.14 ± 24.18	30.74 ± 7.12	9.88 ± 5.02	55.33 ± 10.31
	Mann–Whitney U		U = 23,398, *p* = 0.019, r = 0.09	U = 26,465, *p* < 0.001, r = 0.18	U = 26,090, *p* < 0.001, r = 0.17	U = 25,423, *p* < 0.001, r = 0.15
**Smoking**	Yes	218	53.35 ± 24.19	32.37 ± 7.54	11.28 ± 4.92	57.42 ± 10.81
	No	416	48.10 ± 23.55	30.62 ± 7.22	9.60 ± 5.02	55.10 ± 10.20
	Mann–Whitney U		U = 50,727, *p* = 0.014, r = 0.10	U = 50,863, *p* = 0.012, r = 0.10	U = 54,102, *p* < 0.001, r = 0.16	U = 51,202, *p* = 0.007, r = 0.11
**Earthquake-affected province**	Yes	480	52.01 ± 24.36	31.76 ± 7.25	10.68 ± 5.02	56.15 ± 10.56
	No	154	43.34 ± 21.10	29.56 ± 7.51	8.60 ± 4.81	55.10 ± 10.14
	Mann–Whitney U		U = 45,080, *p* < 0.001, r = 0.16	U = 43,390, *p* = 0.001, r = 0.13	U = 45,685, *p* < 0.001, r = 0.18	U = 39,762, *p* = 0.157, r = 0.06
**Socioeconomic status**	Low	63	50.87 ± 22.66	31.97 ± 7.34	10.83 ± 5.02	57.52 ± 10.51
	Middle	542	49.92 ± 24.03	31.20 ± 7.36	10.13 ± 5.00	56.04 ± 10.38
	High	29	47.59 ± 24.22	30.07 ± 7.81	9.48 ± 5.88	49.66 ± 10.14
	Kruskal–Wallis H		H = 0.48, *p* = 0.786, ε^2^ = 0.001	H = 2.58, *p* = 0.276, ε^2^ = 0.004	H = 2.13, *p* = 0.345, ε^2^ = 0.003	H = 11.40, *p* = 0.003, ε^2^ = 0.018

Values are mean ± SD. U: Mann–Whitney U; r: effect size (z/√N); H: Kruskal–Wallis statistic; ε^2^: epsilon-squared.

**Table 6:** Hierarchical multiple linear regression analysis predicting self-reported TMD symptom severity (FAI score, 0–100; n = 634).

Predictor	Model 1 B (SE)	β	*p*	Model 2 B (SE)	β	*p*	95% CI (Model 2)	VIF
**Constant**	18.35 (6.41)		0.004	−23.19 (8.42)		0.006	−39.72 to −6.65	
**Sex (female)**	17.99 (2.23)	0.330	<0.001	12.89 (2.16)	0.236	<0.001	8.65 to 17.14	1.22
**Age (years)**	0.28 (0.15)	0.075	0.053	0.52 (0.14)	0.141	<0.001	0.25 to 0.80	1.25
**BMI (kg/m^2^)**	0.01 (0.22)	0.003	0.947	−0.05 (0.20)	−0.009	0.798	−0.44 to 0.34	1.20
**SES: low (ref. middle)**	1.64 (3.01)	0.021	0.585	−0.29 (2.80)	−0.004	0.919	−5.79 to 5.22	1.05
**SES: high (ref. middle)**	−2.20 (4.24)	−0.019	0.605	0.95 (3.85)	0.008	0.804	−6.61 to 8.52	1.02
**Chronic disease**	3.21 (3.03)	0.042	0.289	1.88 (2.70)	0.025	0.487	−3.43 to 7.18	1.01
**Psychological complaint**	5.57 (2.60)	0.074	0.033	−0.02 (2.63)	−0.000	0.993	−5.20 to 5.15	1.07
**Smoking**	7.89 (1.87)	0.157	<0.001	3.39 (1.74)	0.068	0.052	−0.03 to 6.82	1.11
**Earthquake-affected province**	8.96 (1.97)	0.161	<0.001	5.49 (1.90)	0.099	0.004	1.75 to 9.22	1.06
**PSS-14**				0.53 (0.14)	0.163	<0.001	0.26 to 0.80	1.51
**JSS**				1.14 (0.19)	0.242	<0.001	0.77 to 1.51	1.31
**TAS-20**				0.31 (0.10)	0.136	0.002	0.12 to 0.51	1.37
**R^2^ (adjusted R^2^)**	0.147 (0.134)			0.292 (0.279)				
**F (df), *p***	11.92 (9, 624), <0.001			21.39 (12, 621), <0.001				
**ΔR^2^, F-change, *p***				0.146, 42.64, <0.001				

B: unstandardized coefficient (FAI points); SE: heteroscedasticity-consistent (HC3) standard error; β: standardized coefficient; CI: confidence interval; VIF: variance inflation factor; SES: socioeconomic status. Coding: sex, female = 1; chronic disease, psychological complaint, smoking, and earthquake-affected province, yes = 1; SES dummies with middle SES as a reference. Missing BMI values (n = 28) were replaced by the median.

## Data Availability

The anonymized dataset and analysis code are available from the corresponding author upon reasonable request.

## Supplementary Materials

The following supporting information can be downloaded at: https://www.mdpi.com/article/10.3390/jcm15218272/s1, File S1: Study questionnaire (Turkish original and English translation). Table S1: Hierarchical regression Model 2 with the TAS-20 subscales instead of the total score (n = 634). Table S2: Sensitivity analyses: standardized coefficients (β) and *p* values for Model 2. Table S3: Cross-tabulation of self-reported TMD symptom severity (FAI) and alexithymia (TAS-20) categories (n = 634).

## Abbreviations

TMD = temporomandibular disorder; TMJ = temporomandibular joint; FAI = Fonseca Anamnestic Index; TAS-20 = 20-item Toronto Alexithymia Scale; DIF = difficulty identifying feelings; DDF = difficulty describing feelings; EOT = externally oriented thinking; PSS-14 = 14-item Perceived Stress Scale; JSS = Jenkins Sleep Scale; DC/TMD = Diagnostic Criteria for Temporomandibular Disorders; REM = rapid eye movement; NREM = non-rapid eye movement; DSM-5 = Diagnostic and Statistical Manual of Mental Disorders, Fifth Edition; BMI = body mass index; VIF = variance inflation factor.
