# Supplementary material for: Associations of Self-Reported Temporomandibular Disorder Symptoms with Sleep Problems, Perceived Stress, and Alexithymia in Young Adults: A Cross-Sectional Study
Source: J Clin Med. 2026 Oct 23;15(21):8272. doi: 10.3390/jcm15218272 (PMC13763754; doi:10.3390/jcm15218272)
Supplement: Supplementary file 1 [file jcm-15-08272-s001.zip › jcm-4600104-supplementary.pdf]

Supplementary Materials

Associations of Self-Reported Temporomandibular Disorder Symptoms with Sleep Problems, Perceived Stress and Alexithymia in Young Adults: A Cross-Sectional Study

Supplementary File S1. Study questionnaire

The online questionnaire (Google Forms) consisted of an informed consent statement, a sociodemographic form developed by the authors, and four validated self-report instruments. The sociodemographic questions are reproduced below (Turkish original followed by English translation). Full item wordings of the standardized instruments are available in the cited validation studies.

#	Turkish (as administered)	English translation
1	Çalışmaya katılım durumunuzu onaylayarak devam ediniz.	Please confirm your participation to continue. (“I agree to participate in the study.”)
2	Yaşınız.	Your age (years).
3	Cinsiyetiniz.	Your sex (female / male).
4	Boyunuz.	Your height (cm).
5	Kilonuz.	Your weight (kg).
6	Doğum yeriniz.	Your place of birth.
7	Ailenin sosyo-ekonomik düzeyi?	Socioeconomic status of your family? (low / middle / high)
8	Kronik hastalığınız var mı?	Do you have a chronic disease? (yes / no)
9	Psikolojik rahatsızlığınız var mı?	Do you have a psychological disorder? (yes / no)
10	Sigara kullanıyor musunuz?	Do you smoke? (yes / no)
11	6 Şubat 2023 depremi esnasında hangi şehirdeydiniz?	In which city were you during the 6 February 2023 earthquake?

Instrument	Items	Response format	Scoring	Turkish validation
Fonseca Anamnestic Index (FAI)	10	Yes (10) / Sometimes (5) / No (0)	Total 0–100; no TMD 0–15, mild 20–40, moderate 45–65, severe 70–100	Arıkan et al., 2024 [44]
Toronto Alexithymia Scale (TAS-20)	20	1 = strongly disagree … 5 = strongly agree	Items 4, 5, 10, 18, 19 reversed; total 20–100; DIF, DDF, EOT subscales; ≤51 / 52–60 / ≥61	Güleç et al., 2009 [45]
Jenkins Sleep Scale (JSS)	4	0 = not at all … 5 = 23–31 days per month	Total 0–20	Duruöz et al., 2018 [47]
Perceived Stress Scale (PSS-14)	14	0 = never … 4 = very often	Items 4, 5, 6, 7, 9, 10, 13 reversed; total 0–56	Eskin et al., 2013 [49]

Table S1. Hierarchical regression Model 2 with the TAS-20 subscales instead of the total score (n = 634)

Predictor	B	SE (HC3)	β	p	95% CI	VIF
Constant	-12.21	8.82		0.167	-29.54 to 5.12	
Sex (female)	11.94	2.16	0.219	<0.001	7.71 to 16.18	1.24
Age (years)	0.59	0.14	0.157	<0.001	0.31 to 0.86	1.27
BMI (kg/m²)	-0.06	0.20	-0.012	0.748	-0.46 to 0.33	1.20
SES: low (ref. middle)	-1.29	2.81	-0.016	0.645	-6.80 to 4.22	1.07
SES: high (ref. middle)	0.98	4.03	0.009	0.808	-6.94 to 8.90	1.03
Chronic disease	1.60	2.68	0.021	0.551	-3.66 to 6.86	1.01
Psychological complaint	-0.90	2.63	-0.012	0.732	-6.08 to 4.27	1.08
Smoking	3.17	1.75	0.063	0.070	-0.27 to 6.61	1.13
Earthquake-affected province	5.68	1.89	0.102	0.003	1.97 to 9.39	1.07
PSS-14	0.47	0.14	0.144	<0.001	0.20 to 0.74	1.53
JSS	1.06	0.19	0.224	<0.001	0.69 to 1.43	1.33
TAS-20 DIF	0.89	0.22	0.224	<0.001	0.47 to 1.32	2.44
TAS-20 DDF	-0.13	0.29	-0.021	0.661	-0.71 to 0.45	2.01
TAS-20 EOT	-0.32	0.23	-0.047	0.162	-0.76 to 0.13	1.08

$R^2 = 0.308$; adjusted $R^2 = 0.292$; $F(14, 619) = 19.67$, $p < 0.001$; ΔR^2 over Model 1 = 0.161. Dependent variable: FAI total score (0–100). Coding as in Table 6.

Table S2. Sensitivity analyses: standardized coefficients (β) and p values for Model 2

Predictor	(i) Main	(ii) PSS-10	(iii) ≥18 y	(iv) No psych.	(v) FAI −item 10	Orig. n=728
Sex (female)	0.236 (<0.001)	0.233 (<0.001)	0.222 (<0.001)	0.245 (<0.001)	0.242 (<0.001)	0.218 (<0.001)
Age (years)	0.141 (<0.001)	0.135 (<0.001)	0.017 (0.638)	0.153 (<0.001)	0.140 (<0.001)	0.017 (0.639)
BMI (kg/m²)	-0.009 (0.798)	-0.007 (0.845)	0.002 (0.951)	0.004 (0.922)	-0.020 (0.588)	-0.001 (0.974)
SES: low (ref. middle)	-0.004 (0.919)	-0.004 (0.899)	0.011 (0.734)	-0.005 (0.884)	0.001 (0.979)	0.012 (0.719)
SES: high (ref. middle)	0.008 (0.804)	0.011 (0.751)	0.014 (0.657)	0.012 (0.722)	0.006 (0.852)	0.014 (0.656)
Chronic disease	0.025 (0.487)	0.023 (0.521)	0.021 (0.522)	0.014 (0.714)	0.020 (0.580)	0.031 (0.344)
Psychological complaint	-0.000 (0.993)	0.002 (0.944)	0.007 (0.823)	—	-0.013 (0.718)	0.002 (0.943)
Smoking	0.068 (0.052)	0.066 (0.060)	0.074 (0.023)	0.049 (0.183)	0.051 (0.152)	0.075 (0.021)
Earthquake-affected province	0.099 (0.004)	0.099 (0.004)	0.077 (0.016)	0.103 (0.005)	0.100 (0.004)	0.073 (0.020)
PSS-14 / PSS-10	0.163 (<0.001)	0.157 (<0.001)	0.169 (<0.001)	0.192 (<0.001)	0.129 (0.004)	0.168 (<0.001)
JSS	0.242 (<0.001)	0.239 (<0.001)	0.246 (<0.001)	0.235 (<0.001)	0.237 (<0.001)	0.245 (<0.001)
TAS-20	0.136 (0.002)	0.141 (0.001)	0.134 (<0.001)	0.129 (0.005)	0.132 (0.003)	0.134 (<0.001)
n	634	634	718	563	634	728
R^2	0.292	0.291	0.290	0.301	0.262	0.285

Values are β (p). (i) main analysis, participants aged 18–40 years; (ii) PSS-10 replaces PSS-14; (iii) all respondents aged ≥ 18 years; (iv) participants with a self-reported psychological complaint excluded; (v) dependent variable = FAI without item 10 (nervousness, 0–90); original sample = all 728 respondents with known age as in the original submission. All models use corrected TAS-20 scoring and HC3 standard errors.

Table S3. Cross-tabulation of self-reported TMD symptom severity (FAI) and alexithymia (TAS-20) categories (n = 634)

FAI category	Non-alexithymic (≤51)	Borderline (52–60)	Alexithymic (≥61)	Total
No TMD	43 (71.7%)	10 (16.7%)	7 (11.7%)	60
Mild	81 (41.5%)	62 (31.8%)	52 (26.7%)	195
Moderate	59 (28.5%)	66 (31.9%)	82 (39.6%)	207
Severe	48 (27.9%)	38 (22.1%)	86 (50.0%)	172
Total	231	176	227	634

Row percentages. $\chi^2(6) = 60.73$, $p < 0.001$.
